# DNA storage: research landscape and future prospects

**DOI:** 10.1093/nsr/nwaa007

**Published:** 2020-01-21

**Authors:** Yiming Dong, Fajia Sun, Zhi Ping, Qi Ouyang, Long Qian

**Affiliations:** Center for Quantitative Biology and Peking-Tsinghua Center for Life Sciences, Peking University, Beijing 100871, China; Center for Quantitative Biology and Peking-Tsinghua Center for Life Sciences, Peking University, Beijing 100871, China; Academician Workstation of BGI Synthetic Genomics, BGI-Shenzhen, Shenzhen 518083, China; Center for Quantitative Biology and Peking-Tsinghua Center for Life Sciences, Peking University, Beijing 100871, China; The State Key Laboratory for Artificial Microstructures and Mesoscopic Physics, School of Physics, Peking University, Beijing 100871, China; Center for Quantitative Biology and Peking-Tsinghua Center for Life Sciences, Peking University, Beijing 100871, China

**Keywords:** DNA storage, information, compilation, sequencing

## Abstract

The global demand for data storage is currently outpacing the world's storage capabilities. DNA, the carrier of natural genetic information, offers a stable, resource- and energy-efficient and sustainable data storage solution. In this review, we summarize the fundamental theory, research history, and technical challenges of DNA storage. From a quantitative perspective, we evaluate the prospect of DNA, and organic polymers in general, as a novel class of data storage medium.

## INTRODUCTION: INFORMATION AND STORAGE

Human civilization went through paradigm shifts with new ways of storing and disseminating information. To survive in the complex and ever-changing environment, our ancestors created utensils out of wood, bone and stone, and used them as media for recording information. This was the beginning of human history [1]. With the development of computer technology, the information age has revolutionized the global scene. Digital information stored in magnetic (floppy disks), optical (CDs) and electronic media (USB sticks) and transmitted through the internet promoted the explosion of next-generation science, technology and arts.

With the total amount of worldwide data skyrocketing, traditional storage methods face daunting challenges [2]. International Data Corporation forecasts that the global data storage demand will grow to 175 ZB or 1.75 × 10^14^ GB by 2025 (in this review, ‘B’ refers to Byte while ‘b’ refers to base pair) [3]. With the current storage media having a maximal density of 10^3^ GB/mm^3^ [4], this will far exceed the storage capacity of any currently available storage method. Meanwhile, the costs of maintaining and transferring data, as well as limited lifespans and significant data losses, also call for novel solutions for information storage [5,6].

On the other hand, since the very beginning of life on Earth, nature has solved this problem in its own way: it stores the information that defines the organism in unique orders of four bases (A, T, C, G) located in tiny molecules called deoxyribonucleic acid (DNA), and this way of storing information has continued for 3 billion years. DNA molecules as information carriers have many advantages over traditional storage media. Its high storage density, potentially low maintenance cost and other excellent characteristics make it an ideal alternative for information storage, and it is expected to provide wide practicality in the future [7].

## OVERVIEW OF DNA STORAGE

### Research history

In 1953, Watson and Crick published one of the most fundamental articles in the history of biology in *Nature*, revealing the structure of DNA molecules as the carrier of genetic information [8]. Since then, it has been recognized that the genetic information of an organism is stored in the linear sequence of the four bases in DNA. In just a decade, many researchers had proposed the concept of storing specific information in DNA [9–11]. However, the concept failed to materialize because the techniques for synthesizing and sequencing DNA were still in their infancy.

In 1988, the artist Joe Davis made the first attempt to construct real DNA storage [12]. He converted the pixel information of the image ‘Microvenus’ into a 0–1 sequence arranged in a 5 × 7 matrix, where 1 indicated a dark pixel and 0 indicated a bright one. This information was then encoded into a 28-base-pair (bp) long DNA molecule and inserted into *Escherichia coli*. After retrieval by DNA sequencing, the original image was successfully restored. In 1999, Clelland proposed using a method based on ‘DNA micro-dots’ like steganography to store information in DNA molecules [13]. Two years later, Bancroft proposed using DNA bases to directly encode English letters, in a way similar to encoding amino acid sequences in DNA [14].

However, these early attempts only stored less than tens of Bytes—a small amount of data with little scalability for practical usages. It was not until the first 10 years of the twenty-first century that the groundbreaking work of Church and Goldman led to the return of DNA storage to mainstream interest [15,16]. Church *et al.* successfully stored up to 659 KB of data in DNA molecules, while the maximal amount of stored data before this work was less than 1 KB [17]. Goldman *et al.* stored even more data, reaching 739 KB. It is worth noting that the data stored in the two studies contained not only texts, but also images, sounds, PDFs, etc., which confirmed that DNA can store a wide variety of data types.

**Figure 1.**
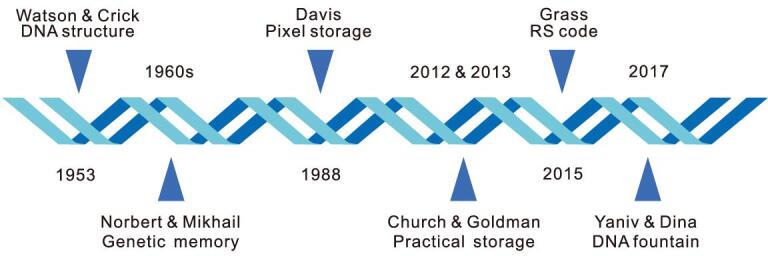
The history of DNA storage. The figure shows seminal publications in the history of research on DNA storage [8,12,15,16,19,20].

Church and Goldman's work led to a research fever of large-scale DNA storage. With increasingly complex compilation methods, the amounts of stored data gradually increased. By the end of 2018, the maximal amount of data stored in DNA exceeded 200 MB, which was stored in more than 13 million oligonucleotides [18]. Along with the development of DNA synthesis and sequencing technologies, new DNA storage methods keep emerging, bringing DNA storage ever closer to practical applications (Fig. 1).

### Self-information of DNA molecules

The capacity of a medium to store information is usually measured by the Shannon information. Since the DNA molecule is a heterogeneous polymer composed of a linear chain of deoxyribonucleotide monomers each adopting one of four bases A, T, C and G, the specific arrangement (i.e. sequence) provides a certain amount of information. According to the definition of Shannon information, the maximal amount of self-information (*H*) that a single base can hold is

$$\begin{eqnarray*}H &=& - \mathop \sum \limits_i^{A,T,C,G} P\!(i\!)\!\log\! P(i\!)\nonumber\\ &\le& \log \mathop \sum \limits_i^{A,T,C,G} \!P( i\! )\ \frac{1}{{P( i\!)}} = \log 4 = 2\ \rm {bit}, \end{eqnarray*}$$

where *P*(*i*) represents the probability of base *i* to occur at any position, and log represents the base 2 logarithm as the bit (binary unit) is usually used as a measurement of digital information [21]. If and only if the four bases are equally likely to occur, that is, *P_i_* = 1/4, each base pair in the DNA molecule can provide the largest information capacity, i.e. 2 bits. The dependence of self-information on base distributions is given in Table 1, where *a* is the ‘probability distribution deviation’, that is, the difference between the frequency at which the base appears and the average frequency of 0.25.

**Table 1.** Probability distribution of bases and the corresponding self-information values.

*a*	*P_A_*, *P_T_ *= 0.25 – *a*	*P_C_*, *P_G_ *= 0.25 + *a*	*I*(X) (bit/base)	*I*(X)/*I*_max_(X)*
0	0.25	0.25	2	100%
0.001	0.249	0.251	1.999988	99.999%
0.005	0.245	0.255	1.999711	99.986%
0.01	0.24	0.26	1.998846	99.942%
0.05	0.2	0.3	1.970951	98.548%
0.1	0.15	0.35	1.881291	94.065%
0.15	0.1	0.4	1.721928	86.096%
0.2	0.05	0.45	1.468996	73.450%
0.24	0.01	0.49	1.141441	57.072%

*I_max_(X) = 2 bit/base.

By converting the 2 bit/base to physical density, we obtain

$$\begin{eqnarray*}\rho &=& \frac{{2\ \rm{bit}}}{{1\ \rm{base} \times \ 325\frac{\rm {Dalton}}{\rm {base}} \times \ 1.67 \times {{10}^{ - 24}}\frac{g}{\rm{Dalton}}}}\nonumber\\
&=& 3.69\ \times {10^{21}}\ \frac{\rm {bit}}{g}\nonumber\\
&=& \ 4.61\ \times {10^{20}}\frac{\rm {Byte}}{g} \approx 460\frac{\rm {EB}}{g},\end{eqnarray*}$$

where *ρ* represents density, 1 EB = 10^18^ B (in this paper, the data storage unit has a radix of 10^3^ instead of 1024) and the remaining unit conversion values are derived from ref. [19].

Additional restrictions on the sequence of DNA molecules will further reduce its Shannon information capacity. For example, Erlich *et al.* estimated a Shannon information capacity of ∼1.83 bits per base under intrinsic biochemical constraints and technical limitations of DNA synthesis and sequencing procedures [19].

### Mutual information and channel capacity

In addition to the self-information carried by DNA molecules, mutual information between channel inputs and outputs is also an important factor in determining information capacity [21]. Mutual information measures the fidelity with which the channel output Y = {*y_j_*|A, T, C, G} (i.e. the readout of a DNA by sequencing) represents the channel input X = {*x_i_*|A, T, C, G} (i.e. the preset DNA sequence):

$$\begin{eqnarray*}I\!({X;Y}\!) &=& \mathop \sum \limits_i^{A,T,C,G} \mathop \sum \limits_j^{A,T,C,G} P\!\left( {{x_i}{y_j}} \right)I\!\left( {{x_i}\!\,\,{y_j}}\! \right)\nonumber\\ &=& \mathop \sum \limits_i^{A,T,C,G} \mathop \sum \limits_j^{A,T,C,G} P( {{x_i}{y_j}})\log \frac{{\!P({x_i}|{y_j})}}{{P\!( {{x_i}}\!)}}, \end{eqnarray*}$$

and we have

$$\begin{eqnarray*}
I\!({X;Y}\!) &=& H(X) - H\!( {X{\rm {|}}Y}\! ) \le H( X ).
\end{eqnarray*}$$

For DNA molecules, if each of the four bases corresponds exactly to itself, then *H*(X|Y) = 0, *I*(X; Y) = 2 bit/base, and the average mutual information in the transmission is equal to the source entropy, which gives the upper limit of the amount of information transmitted. However, information may be distorted in the process of writing and reading DNA sequences, causing mismatches between the input set X and the output set Y, which reduces the average mutual information during transmission. For example, if each base corresponds to the other three bases except itself with a probability of 1/10, then

$$\begin{eqnarray*}H\!\!\ \left( {Y{\rm {|}}{x_i}} \right) &=& H\!\! \left( {\frac{1}{{10}},\frac{1}{{10}},\frac{1}{{10}},\frac{7}{{10}}} \right)\nonumber\\
&=& 1.35679\frac{\rm {bit}}{\rm {base}}.\end{eqnarray*}$$

Assuming that the four bases entered are equally probable, we have

$$\begin{eqnarray*}
H\!\! \left( {Y{\rm {|}}X} \right) &=& \ 4\cdot\frac{1}{4}\cdot H\!\!\ \left( {Y{\rm {|}}{x_i}} \right)= 1.35679\frac{\rm {bit}}{\rm {base}},
\end{eqnarray*}$$

while

$$\begin{equation*}H\!\!\ \left( X \right) = \ H\!\!\ \left( Y \right) = \ 2\ \frac{\rm {bit}}{\rm {base}}.\end{equation*}$$

The joint entropy of X and Y is

$$\begin{eqnarray*}H ( {XY}) &=& H( X ) + H ( {Y{\rm {|}}X})\nonumber\\
&=& 3.35679\frac{\rm {bit}}{\rm {base}},\end{eqnarray*}$$

and the average mutual information is

$$\begin{eqnarray*}I\! ( {X;Y}\!) &=& H\!( X) + H\!( Y) - H\! ( {XY})\nonumber\\
&=& 0.64321\frac{\rm {bit}}{\rm {base}}.\end{eqnarray*}$$

**Figure 2.**
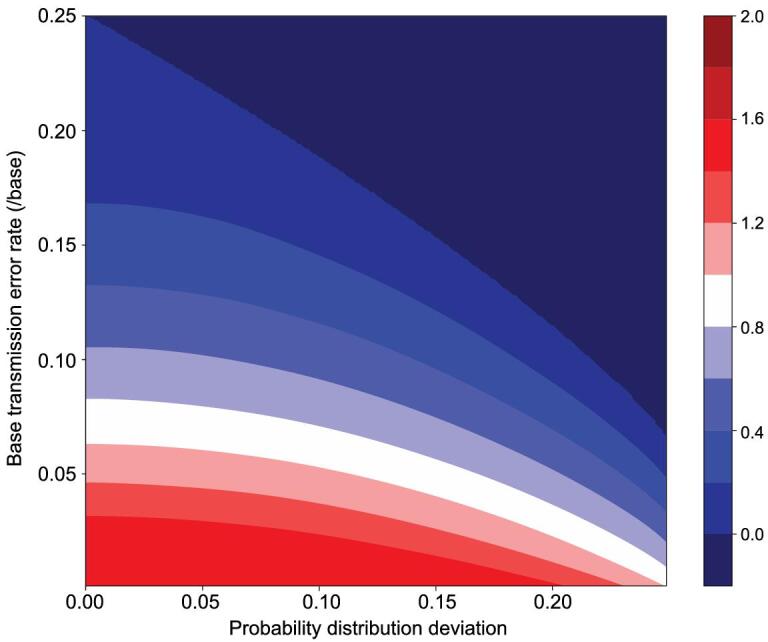
The relationship among the average mutual information transmitted by DNA, the probability distribution deviation of bases and the base transmission error rate. Color indicates the average mutual information values.

Thus, the distortion of the base readout greatly reduces the utility of information transmission in DNA. Table 2 shows the average mutual information at different transmission error rates *m_i_* (the probability that one base is incorrectly read out as one of the other three bases), assuming 2 bit/base inputs. Figure 2 gives the variation of the average mutual information as a function of the input base bias and the transmission error rate.

**Table 2.** Base transmission error rate and the corresponding mutual information.

*mi*	0.001	0.005	0.01	0.02	0.05	0.1	0.2
*I*(X;Y) (bit/base)	1.9658	1.8639	1.7581	1.5774	1.1524	0.64321	0.0781
*I*(X;Y)/*I*_max_(X;Y)*	98.29%	93.19%	87.91%	78.87%	57.62%	32.16%	3.95%

*I_max_(X; Y) = 2 bit/base.

More generally, the channel transmission characteristics of DNA molecules can be defined by a 4 × 4 transfer matrix *T* [21]

$$\begin{equation*}XT = Y,\end{equation*}$$

where *X* is the input set and *Y* is the output set, and *T* can be expanded as:

$$\begin{eqnarray*}T = \left[ {\begin{array}{@{}*{2}{c}@{}} {\begin{array}{@{}*{2}{c}@{}} {{P_{AA}}}&\quad{{P_{AT}}}\\ {{P_{TA}}}&\quad{{P_{TT}}} \end{array}}&\quad{\begin{array}{@{}*{2}{c}@{}} {{P_{AC}}}&\quad{{P_{AG}}}\\ {{P_{TC}}}&\quad{{P_{TG}}} \end{array}}\\ {\begin{array}{@{}*{2}{c}@{}} {{P_{CA}}}&\quad{{P_{CT}}}\\ {{P_{GA}}}&\quad{{P_{GT}}} \end{array}}&\quad{\begin{array}{@{}*{2}{c}@{}} {{P_{CC}}}&\quad{{P_{CG}}}\\ {{P_{GC}}}&\quad{{P_{GG}}} \end{array}} \end{array}} \right],\ \end{eqnarray*}$$

where *P_ij_* refers to the probability that the input base *i* is received as base *j* after channel transmission. If

$$\begin{equation*}\left\{\!\! {\begin{array}{@{}*{1}{c}@{}} {\ {P_{ij}} = \ 1,\quad i\ = \ j}\\ {\ {P_{ij}} = \ 0,\quad i \ne j} \end{array}} \right.,\end{equation*}$$

the above transfer process corresponds to the passage of information through an ideal channel. In reality, the values of *P_ij_* can be obtained for a specific storage method through systematic experimentation. We can therefore obtain

$$\begin{equation*}H( {Y{\rm {|}}{x_i}}\!) = \mathop \sum \limits_j^{A,T,C,G} H({P_{ij}}\!).\end{equation*}$$

If we denote by *P_i_*(*i* = A, T, C, G) the frequency of each base in a channel input, then the corresponding frequency distribution in the output *Y*, as well as the average mutual information, is completely determined by *P_i_* and the transfer matrix *T*

$$\begin{equation*}\ P_i^{\prime} = \mathop \sum \limits_j^{A,T,C,G} {P_j}\cdot{P_{ji}}.\end{equation*}$$

Therefore, we can obtain

$$\begin{eqnarray*}H\!\!\ \left( Y \right) &=& \mathop \sum \limits_i^{A,T,C,G} H(P_i^{\prime}), H\!\! \left( {Y{\rm {|}}X} \right)\nonumber\\ &=& \mathop \sum \limits_i^{A,T,C,G} {P_i}\mathop \sum \limits_{j = 1}^{A,T,C,G} H({P_{ij}}),\end{eqnarray*}$$

and the average mutual information is

$$\begin{eqnarray*}I({X;Y} ) &=& H( Y )-H( {Y{\rm {|}}X} )\nonumber\\
&=& \mathop \sum \limits_i^{A,T,C,G} H\left(\mathop \sum \limits_j^{A,T,C,G} {P_j}\cdot{P_{ji}}\right)\nonumber\\
&&- \mathop \sum \limits_i^{A,T,C,G} {P_i}\mathop \sum \limits_{j = 1}^{A,T,C,G} H({P_{ij}}).\end{eqnarray*}$$

Due to the non-negative nature of the entropy function, the average mutual information can only be maximized when the latter term is 0. This requires that all *P_ij_* values be either 0 or 1, i.e. *X* and *Y* form a strict one-to-one mapping relationship. It is not necessary for each base to correspond to itself, though. For example, if all A in the DNA molecule become T after channel transmission and T→C, C→G, G→A, the maximal mutual information can also be achieved. In practice, this method is cumbersome and unnecessary. However, this approach may have potential uses in information encryption [22].

For a specific storage method with its measured transfer matrix *T*, one may find the input base probability distribution that generates the highest channel capacity [23]

$$\begin{equation*}C\ = \begin{array}{@{}*{1}{c}@{}} {P\!( X )}\\ {\rm {max}} \end{array}\{ {I\!( {X;Y}\! )} \},\end{equation*}$$

which requires

$$\begin{equation*}\ \frac{{\partial\! I\!( {X;Y}\! )}}{{\partial\! {P_i}}} = 0.\end{equation*}$$

After substituting the previously obtained expression for *I*(X; Y), the best input probability distribution can be obtained by calculation.

In addition to mismatches, common errors in synthesis and sequencing include insertions and deletions, collectively called indels. Generally, the impact of indels on information storage is much greater than that of mismatches, since the loss or gain of consecutive sequences may nullify the entire DNA molecule. In next-generation sequencing such as Illumina, indels occur less than 1% as frequently as substitutions do. However, single-molecule sequencing has been reported to be prone to indel errors [24]. Indels in DNA storage correspond to ‘erasure channels’ in the field of information science. Theory on this subject is still under active development. Various models of erasure channels have been established. We refer the readers elsewhere (e.g. ref [25]) without elaboration here.

## IMPLEMENTATION OF DNA STORAGE

**Figure 3.**
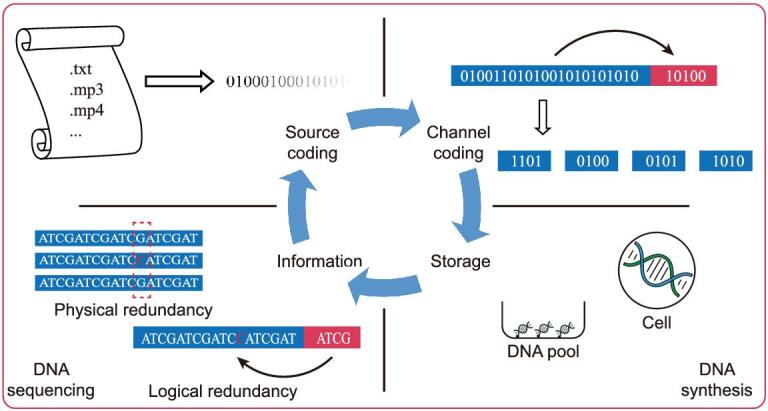
Flow of information in DNA-based information storage. Top left: source coding, i.e. converting information into binary code (or other radix) series. Top right: channel coding, i.e. data error detection/correction coding, providing an error correction/error detection capability by providing additional bits of redundancy. Bottom right: information storage. After the desired DNA molecule is synthesized, it can be stored *in vitro* or *in vivo*. Bottom left: information readout. Each part will be detailed in the text.

Figure 3 summarizes the general workflow of the DNA information storage process.

### Source coding

In order to use DNA molecules for information storage, information must first be converted into a sequence of four bases in the DNA molecule. In general, each base is equivalent to a quaternary number, corresponding to two binary digits. Obviously, any digital information can be encoded into the DNA molecule by a simple conversion. This applies to all types of data that can be stored on a hard drive.

In the field of information science, different data types are processed using different encoding and compression algorithms [23]. Here, we take the classic text-file format as an example to introduce the various compilation methods of DNA storage. In the first attempt by Bancroft *et al.*, English letters were directly encoded by base triplets in a manner like the amino acid codon table, for example, ‘AAA’ represents the letter ‘A’ [14]. Interestingly, they only used three bases to form a ‘ternary digit’, while G was reserved for sequencing primers. The method ignored capitalization because three bases can produce a coding space of only 3^3^ = 27 elements, which is just enough to encode 26 letters. And, by the same reason, this encoding scheme does not apply to other data types.

A pioneering study by Church *et al.*, as the first big volumne DNA storage work, used a more scalable approach. They first converted different files into binary sequences in the *HTML* format and then converted these into DNA sequences [15]. In comparison, Goldman *et al.* applied the Huffman coding scheme in the first step, which employs ternary instead of binary conversion. Huffman coding simultaneously compresses the data and this is the first DNA storage study in which data compression algorithms were used.

In fact, data compression is essential when scaling DNA storage to larger data volumes. For text files, many lossless data compression algorithms exist that greatly reduce the space required to store them. The lower bound of the storage space in a lossless compression scheme is defined by Shannon's first theorem. If the source entropy of a discrete memoryless stationary source is *H*(*X*), using the *r*-ary symbol to encode the *N*-time extended source symbol sequence of the source in variable length, there must be a unique distortion-free and decodable code [21], with the average code length *L* satisfying

$$\begin{equation*}\frac{{H\!\left( X \right)}}{{\log\!\! \ r}} \le \frac{L}{N} < \frac{{H\!\left( X \right)}}{{\log\!\!\ r}} + \frac{1}{N}.\end{equation*}$$

The text files currently stored in the DNA molecules are treated as memoryless sources (i.e. there is no correlation between adjacent letters, *N* = 1). When binary encoding (*r* = 2) is used, the average code length *L* satisfies

$$\begin{equation*}H\!\left( X \right) \le L < H\!\left( X \right) + 1.\end{equation*}$$

Intuitively, the average code length of each symbol in the code cannot be less than the source entropy

$$\begin{equation*}H\ = \ - \mathop \sum \limits_i p( i\! )\log\! p( i\! ),\end{equation*}$$

where *i* represents each letter in the text file and *p*(*i*) is the frequency at which it appears. The available algorithms for text compression include Huffman coding, arithmetic coding, dictionary coding, etc., among which Huffman coding is the most commonly used in the field of DNA storage. This is a variable-length code that uses shorter codes for high frequency letters and longer codes for low frequency letters to reduce the average code length of the text file. The Huffman coding algorithm is readily applicable to any text file and is compatible with special characters.

It is worth mentioning that, for a particular language, it is possible to encode a piece of text with a shorter code length. In English, for example, the frequency of the 26 letters in a typical text varies greatly. If they are assumed to be statistically independent, they are equivalent to a discrete memoryless source. Statistical analyses revealed the average source entropy of English texts is [26]

$$\begin{equation*}{H_s} = \ - \mathop \sum \limits_{i\ = \ 1}^{27} p(i )\log\!\ p( i )\ = \ 4.02\ \left( {\frac{\rm {bit}}{\rm {letter}}} \right).
\end{equation*}$$

However, in a text context, English letters are in fact not statistically independent. Shannon studied the English text as an *n*^th^-order Markov source. For *n* → ∞, he obtained the statistical inference value [26]

$$\begin{equation*}\ {H_\infty } = \ 1.4\ \left( {\frac{\rm {bit}}{\rm {letter}}} \right).\end{equation*}$$

${H_\infty }$ is called the limit entropy. For any finite *n*, it is possible to compress information to reach density *H_n_*(${H_\infty } < {H_n} < {H_s}\!)$ by considering the context dependencies among letters.

### Channel coding

Information distortion often occurs during transmission [21]. For DNA molecules, errors may occur during synthesis, replication and sequencing. There are two ways to recover raw data despite information distortion: physical redundancy and logical redundancy. Physical redundancy entails increasing the copy number of DNA molecules that encode the same information. For example, Goldman *et al.* used 4-fold redundant DNA molecules to store information in their initial attempts, i.e. in each short DNA molecule of 100 bp long, the first 75 bp overlapped with the previous molecule and the last 75 bp overlapped with the next molecule [16]. Previous work by Nozomu *et al.* used different sequences to encode the same information. In the process of mapping the binary 0–1 sequence to DNA bases, a binary number was shifted each time and the corresponding base sequences were obtained. As a result, they were able to encode the same information using four different base sequences [27].

Sequencing coverage also contributes to physical redundancy. In the initial work of Church *et al.*, the sequencing coverage was 3000× [15]. However, physical redundancy is not sufficient for achieving lossless data transmission. The work of Goldman and Church failed to completely restore all the information. Church *et al.* found a total of 22 errors in the sequencing results [15] and Goldman *et al.* also obtained sequences that cannot be automatically recovered [16]. In addition, for large data volumes, physical redundancy imposes a dramatic increase in costs.

**Figure 4.**
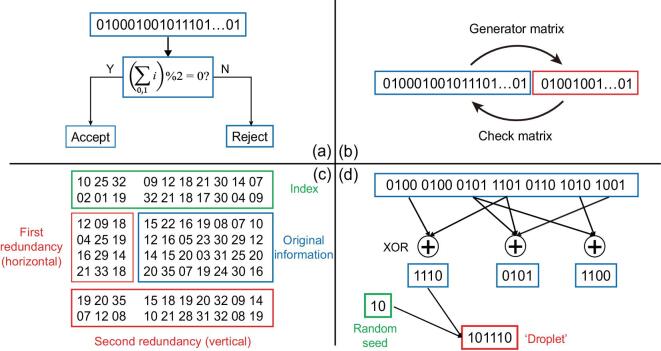
Illustrations of channel coding for DNA storage. (a) Hamming code, which can only be used to check one error. (b) Linear block code. (c) RS code. Shown here is the two-round RS code used by Grass *et al.* [20]. (d) Fountain code. Shown here is the LT code used by Erlich *et al.* [19].

Another way to correct errors is by logical redundancy—a method widely used in the communication field. The general idea of logical redundancy is to add extra symbols, called ‘check symbols’ or ‘supervised symbols’, in addition to the symbols encoding information. When the information symbols are incorrect, the check symbols can be used to detect or correct errors so that the information can be accurately recovered (Fig. 4).

The most commonly used error correction code is the linear block code (Fig. 4b). Specifically, if a group of information symbols has a length of *k*, a check symbol of length *r* can be added using a specific generator matrix to obtain a linear block code with a code length of *n = k + r*. Once the generator matrix is selected for a set of codes, the pairing between the information symbols and the check symbols determines whether a codeword is legal or not. The apparent coding efficiency of this code is *k/n* and the error correction capability scales with *r/n =* 1 – *k/n*. Thus, there is a trade-off between the coding efficiency and the error correction capability.

The most basic class of linear block codes is the Hamming code (Fig. 4a). Simple as it is, only one error can be detected in each group of code words. Due to its obvious limitations, the Hamming code has not been used for DNA storage. Another class of linear block code is called the cyclic code, by which each group of codewords is still legal after one cyclic shift. The most widely used type of cyclic code is the Bose–Chaudhuri–Hocquenghem (BCH) code, which is a code class that can correct multiple random errors based on the Galois binary field and its extension. To avoid crossover between the information symbol and the check symbol, one can use a generator polynomial to get a special BCH code, which is called a system code [21].

Quantitative assessments can be performed to compare the usefulness of physical redundancy and logical redundancy. For second-generation sequencing, several studies on DNA storage in recent years have pointed out that the total error rate in the synthesis–storage–sequencing process (equivalent to channel transmission) is about 1% [28,29]. Assuming misread events are independent and identically distributed, their total number follows the Poisson distribution. For instance, for a DNA molecule of 128 bp in length, the probability of any error occurring is

$$\begin{equation*}{P_E} = 1 - \frac{{{e^{ - 128 \times \ 0.01}} \times {{\left( {128 \times 0.01} \right)}^0}}}{{\ 0!}} = \ 0.722.\end{equation*}$$

If 3-fold physical redundancy is used for error correction, the error becomes incorrectible when more than two of the three copies are misread for the same base at the same site. Therefore, the probability of an uncorrected error is

$$\begin{equation*}\mathop \sum \limits_{i\ = \ 0}^1 C_3^i\cdot{0.99^i}\cdot\ {\left( {1 - 0.99} \right)^{3 - i}} = \ 0.000298,\end{equation*}$$

at any base. For the 128-bp DNA molecule, the probability of any error occurring is

$$\begin{eqnarray*}{P_E} &=& 1 - \frac{{{e^{ - 128 \times 0.000298}} \times {{0.0128}^0}}}{{0!}}\nonumber\\ &=& 0.03742568.\end{eqnarray*}$$

Now let us turn to logical redundancy. We will use the (255, 207) BCH code as an example (note that this corresponds to the above 128-bp DNA molecule), which can correct six errors in each group of 255-bit symbols. Still using the overall error rate of 1% per base, the code fails to correct all errors only when at least seven errors occur in a group of code words, which has a probability

$$\begin{eqnarray*} {P_E} = 1 - \mathop \sum \limits_{i = 0}^6 \frac{{{e^{ - 2.55}} \times {{2.55}^i}}}{{i!}} = 0.016.\end{eqnarray*}$$

**Figure 5.**
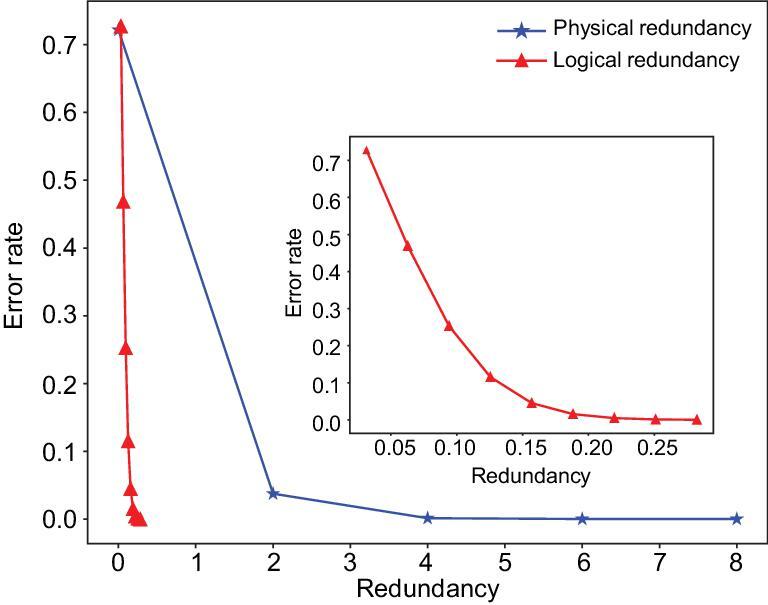
The error correction capacity of coding systems with different levels of physical and logical redundancies. The ‘error rate’ on the *y*-axis refers to the probability of not being able to correct all the errors. Blue line: the effect of physical redundancy on error correction capacity (taking 128-bp DNA as an example). Red line: the effect of logical redundancy on error correction capacity. Here, an original BCH code with code length *n* = 255 is used as an example. Inset: magnified view of logical redundancy.

It can be seen that a logical redundancy of <20% already suppresses error rates to a similar extent as a physical redundancy of 200% does. Shannon's second theorem states that, for a discrete memoryless channel with capacity *C* and a discrete source with entropy per second *R*, if $R \le C$, then, as long as the code length *n* is large enough, an encoding rule and a corresponding decoding rule can always be established to make the average error probability *P_E_* arbitrarily small. Figure 5 compares varying degrees of physical and logical redundancy and their error-correction capabilities.

The Reed-Solomon (RS) code that has been applied in DNA storage is a special non-binary BCH code, which has been widely used in fiber, satellite and deep-sea communication, etc. [21]. Grass *et al.* used the RS codes generated on the Galois Field GF (47) for error correction [20]. Notably, they added two rounds of RS codes, called the ‘inner code’ and the ‘outer code’, respectively, to map the information symbols along orthogonal directions (Fig. 4c). The outer code also mapped the indices. This type of coding is optimized to correct bursts of errors, such as in the case of consecutive base losses, i.e. sequence degradation. In addition, RS codes were included in the ‘DNA fountain’ system used by Erlich *et al.*, where they were not used for error correction, but for detecting and discarding erroneous sequences [19].

By contrast, fountain coding uses a completely different framework than linear block codes, amounting to a codeless erasure code. The basic idea is to group the signal sources into smaller packets. After obtaining an adequate number of packets, the original information can be successfully restored (Fig. 4d). The main advantage of the fountain code is its extremely low redundancy and it can handle ‘erase’ (deletion and insertion of bases) errors. Erlich *et al.* used the classic Luby Transform Code in the fountain code, i.e. the LT code. If DNA molecules are lost to varying degrees, the LT code can still handle it well through detailed design. Currently, the fountain code may be the only error-correction code in the field of DNA storage that can robustly deal with the loss of DNA molecules. The success of commercial LT codes for digital information (achieving a decoding failure rate <10^−8^ with <5% redundancy [30]) has highlighted its potential for DNA storage.

### Encoding information in DNA sequences

After being converted to a binary (or other radix) sequence, the information needs to be transformed into base sequences in DNA. For binary data, the most intuitive conversion is representing 2 bits with one base. The correspondence can be set arbitrarily to control the base compositions in a specific DNA molecule. Furthermore, this method provides the maximal information storage capacity. However, it may result in sequences that are difficult to manipulate, such as long tracts of homopolynucleotides that are error-prone in high-throughput sequencing [31].

Much of the previous work was focused on solutions to this problem. Church *et al.* used one base to represent a single binary digit (i.e. A, C = 0; G, T = 1), so that alternative bases can be adopted to avoid homopolynucleotide tracts [15]. However, the low information density prevented its use in later studies. Goldman *et al.* pioneered in a ternary base conversion table that allows each base to represent a ternary number depending on the previous base [16]. This approach absolutely avoids homopolynucleotide tracts without compromising information density. In the fountain coding scheme by Erlich *et al.*, a single base can still correspond to two binary digits, with unqualified sequences discarded altogether in transmission [19]. They further analysed the constraint on the GC content of DNA molecules as it affects the stability of DNA molecules, the substitution and indel error rates during sequencing, and the dropout rates in PCR amplifications, which were also emphasized in other work [32]. An appropriate GC content close to 50% can be obtained through proper base encoding methods as well as by sequence screening—that is, selecting DNA molecules with appropriate GC ratios to store information while discarding molecules with unreasonable GC contents. In the sequence screening scheme, Erlich *et al.* gave an estimate of 1.98 bits/nt for the maximal coding capacity of DNA storage considering the effects of homopolymers and GC contents, although the latter contributes a comparatively small reduction [19].

### Information density of DNA storage

As shown in the previous section, the upper limit of the information storage density of DNA has been calculated to be about 4.606 × 10^20^ Bytes/g, but a more practical indicator is the volumetric density. In the initial work of Church *et al.* [15], the bulk density of DNA molecules was approximated to the density of pure water, which gave an information density of 4.606 × 10^17^ Bytes/mm^3^. In comparison, the information storage density of classic media, such as flash drives, optical tape and hard disks, is of the order of 10^9^ Bytes/mm^3^ [4,5].

However, the estimate was made under ‘ideal conditions’, ignoring many practical factors. First, the theoretical bulk density can hardly be reached, as DNA molecules need to be stored in specific environments to prevent degradation. For example, most *in vitro* DNA storage studies were based on short DNA oligonucleotides (oligos) in a DNA pool, which was dissolved in dilute solution. Second, physical and logical redundancies reduce the actual information density to various extents. Third, a certain length of index is needed in the DNA molecules to provide addresses, which are themselves not available for storing information.

Here, we briefly analyse the indexing demand of *in vitro* DNA oligo storage. Due to technical bottlenecks in the current DNA synthesis process, most studies to date have used 150- to 250-bp oligos as storage units. Since DNA oligos are fully mixed in a library, a unique index needs to be assigned to each oligo encoding unique information. Table 3 shows the length of the index required in a 200-bp molecule when storing different amounts of data. When the length of the index in this sequence is *k* bp, the number of indexable molecules is 4*^k^* and the number of bits used to store information is 400–2*k* per molecule. Therefore, the total storage capacity of the oligo pool is

$$\begin{equation*}Q\left( k \right)\ = \left( {400 - 2k} \right)\ \cdot{4^k}\ \rm {bit}.\end{equation*}$$

**Table 3.** Index length required to store different amounts of data with 200-bp DNA molecules.

Data amount	1 Byte	1 KB	1 MB	1 GB	1 TB	1 PB	1 EB	1 ZB
Index length (bp)	0	3	8	13	18	23	28	33
Index ratio*	0	1.5%	4%	6.5%	9%	11.5%	14%	16.5%

*Index ratio = *L*(index)/*L*(molecule).

In reality, it is almost impossible to store ZB orders of data in a single DNA oligo library. For example, the dilute solution condition, as is required for efficient information retrieval and amplification, is hardly met, with 4^33^ ≈ 10^20^ molecules dissolved in a few liters of solution. Another constraint is imposed by the free diffusion of DNA oligos in solution. Although, in the 100 base pair range, the diffusion coefficient of DNA oligos can be higher than 10 μm^2^/s, the Brownian motion of oligos could not traverse a significant portion of the reaction system in a reasonable reaction time to enable searching of the probes for random access of information, especially in large libraries. Our crude calculations suggest an upper limit of PB information in a 1-liter reaction system. Lastly, the theoretical indexing limit should not be saturated to ensure sufficient specificity of indices against probes. One possible solution for the storage of large data volumes is to use physically separated DNA pools. This has not been explored yet, due to the extremely limited amount of information that has been stored in DNA so far. However, as DNA storage comes close to real practice, rigorious systems design such as this will be needed.

Finally, as mentioned in the previous sections, intrinsic limits of DNA synthesis and sequencing technologies impose constraints on the DNA sequences that could code information reliably, which reduces the information storage density of DNA molecules (e.g. Fig. 2).

**Figure 6.**
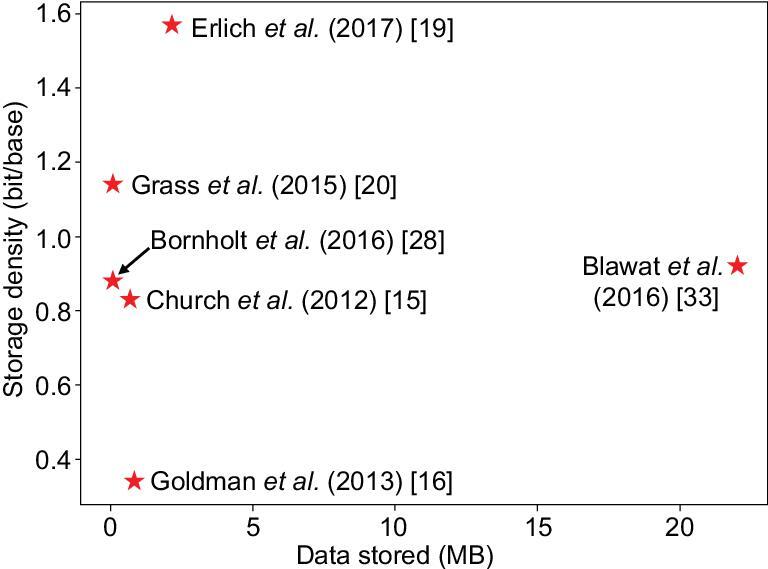
Amounts of data stored and storage densities achieved in major DNA data storage studies. The storage density refers to the effective density, i.e. the total amount of information stored divided by the total number of bases used (number of oligonucleotides × number of bases per oligonucleotide molecule). The *x*-axis shows the total amount of data stored [15,16,19,20,28,33].

Figure 6 shows the amounts of data stored and the data storage densities achieved in major DNA data storage publications since 2012.

## TECHNICAL ASPECTS AND PRACTICAL CONSIDERATIONS

### DNA synthesis and assembly technology

The past few decades have witnessed the rapid development of DNA synthesis and assembly technologies, which laid the groundwork for the advancement of novel fields and technologies including DNA information storage.

The first generation of DNA synthesis techniques are based on solid-phase phosphoramidite chemistry [34,35]. The main advantage of this method is its high accuracy, albeit with a high cost and a low throughput. Moreover, for the consideration of sequence integrity and synthesis efficiency, the product length is limited to 150–200 bp. The second-generation, array-based DNA synthesis is a technique for synthesizing DNA using a series of electrochemical techniques on microarray chips. In each cycle, nucleotides are conjugated to DNA strands at specific locations of the chip, allowing simultaneous elongation of a heterogeneous pool of oligos [36]. Array-based DNA synthesis significantly improved the speed, efficiency and cost-effectiveness of DNA synthesis. In particular, the 10^6^ parallel throughput achieved on current state-of-the-art second-generation platforms increases the total speed of synthesis to a few kilo bases per second. The third-generation DNA synthesis techniques are based on enzymatic synthesis. Although still in their infancy, they are expected to dramatically reduce the time and cost of DNA synthesis. Lee *et al.* gave an estimate of 40 s/cycle, which is six times as fast as phosphoramidite synthesis, and a projected reduction in cost by several orders of magnitudes once their terminal deoxynucleotidyl transferase (TdT) enzymatic reaction system is miniaturized [37].

In addition to DNA synthesis technology, DNA ligation and assembly technologies will provide powerful support for DNA information storage and in particular long-chain DNA storage. At present, commonly used DNA amplification, ligation and assembly techniques include PCR [38], loop-mediated isothermal amplification (LAMP) [39], overlap-extension PCR (OE-PCR) [40], circular polymerase extension cloning (CPEC) [41], InFusion technology [42], sequence- and ligation-independent cloning (SLIC) [43], restriction enzyme digestion and ligation [44], as well as Gibson [45] and Golden Gate assembly [46–48].

### DNA sequencing technology

Since the invention of the Sanger sequencing method in 1977, DNA sequencing has developed into a fully fledged technology, with its cost dropping by 100 000 times in recent years [49]. Based on the underlying mechanisms, DNA sequencing is generally divided into three generations: Sanger sequencing, high-throughput sequencing/Next Generation Sequencing (NGS) and single-molecule sequencing.

The first generation of sequencing technology is based on Sanger's double-deoxygenation termination sequencing combined with fluorescent labeling and capillary array electrophoresis [50]. Currently, automated first-generation DNA sequencing is still widely used.

The core idea of NGS is large-scale parallel sequencing, which enables the simultaneous sequencing of hundreds of thousands to millions of DNA molecules with short read lengths. The available platforms include Roche/454 FLX, Illumina/Solexa Genome Analyzer, HiSeq and ABI/Applied Biosystems SOLID system, Life Technologies/Ion Torrent semiconductor sequencing, etc. [51–54]. NGS has raised the sequencing throughput from 100 Kb to the orders of Gb and Tb, and reduced the cost of sequencing at a rate four times that predicted by Moore's Law [49].

The Helicos/HeliScope single-molecule sequencer [55], Pacific Biosciences SMRT technology [56,57], Oxford Nanopore Technologies nanopore single-molecule technology [58,59] and single-cell genomic sequencing technology [60] are considered third-generation single-molecule sequencing technologies. Besides removing the dependence on PCR amplification, third-generation sequencing has managed to significantly increase the read length and raise the read speed. The cost and accuracy are currently less than satisfactory but are expected to improve with further technological development, making it more practical for the purpose of DNA information storage [52–60]. Table 4 compares performance of typical sequencing techniques from the three generations.

**Table 4.** Comparison of three generations of DNA sequencing technology [50,52–54].

Sequencing technology	First generation (Sanger)	Second generation (Illumina)	Third generation (ONT nanopore)
Cost (per Kb)	$1–2	$10^–5^–10^–3^	$10^–4^–10^–3^
Error rate	0.001–0.01%	0.1–1%	∼10%
Sequencing length	1 Kb	25–150 bp	200 Kb
Read speed (per Kb)	∼10^–1^ h	∼10^–7^–10^–4^ h	10^–7^–10^–6^ h
Sequencing throughput	1 Kb	10^8^–10^12^ bp	10^9^–10^13^ bp

### Cost of DNA data storage

Compared to traditional data storage methods, DNA storage has significantly lower storage maintenance costs. For example, if a data center stores 10^9^ G data on tape, it will require as much as $1 billion and hundreds of millions of kilowatts of electricity to build and maintain for 10 years [5]. DNA storage can reduce all these expenses by 3 orders of magnitude [5]. Nevertheless, the cost of DNA synthesis can be significant and it will become a limiting factor for DNA storage to commercialize. At the current cost of ∼$10^−4^/base [61] and a coding density of 1 bit/base, a conservative estimate of the write cost is $800 million/TB, while tape costs about $16/TB [62]. On the other hand, the read cost achieved by current sequencing technologies is orders of magnitude smaller, at ∼$0.01–1 million/TB [63]. However, it is expected that the cost of DNA synthesis and sequencing will continue to decrease in the future, and new techniques and methods will be applied to DNA storage [52].

### The age limit of DNA storage

DNA molecules naturally decay with a characteristic half-life [64,65], leading to a gradual loss of stored information. The half-life of DNA highly correlates with temperature and the fragment length. For example, Allentoft concluded that a DNA molecule of 500 bp has a half-life of 30 years at 25°C, which extends to 500 years for a fragment of 30 bp. Interestingly, fossils provide empirical evidence of DNA’s stability over thousands of years [65]. In this case, stability is significantly improved by low temperatures and waterproof environments [65]. Indeed, at −5°C, the half-life of the 30-bp mitochondrial DNA fragment in bone is predicted to be 158 000 years [65]. Some studies have suggested that DNA can be placed in the extremely cold regions of Earth or even on Mars for millennium-long storage. Other studies have explored packaging materials for DNA molecules and have demonstrated impressive stability [66,67]. Grass *et al.* encapsulated solid-state DNA molecules in silica and showed that they had better retention characteristics than pure solid-state DNA and DNA in liquid environments [20]. Judging by first-order degradation kinetics, they concluded that it could survive for 2000 years at 9.4°C or 2 million years at −18°C, surpassing all potential quantitative data storage materials invented to date. It is reasonable to expect a long lifetime for data stored in DNA even at room temperature, which makes DNA storage especially suited for cold data with infrequent access. Further research may extend the lifetime of DNA storage over the duration of human civilization with minimal maintenance.

### *In vivo* DNA storage

Most DNA storage attempts to date were done *in vitro*. However, the genomic DNA of living cells has become an ideal medium for information storage due to its durability and bio-functional compatibility. Its advantages are becoming more obvious with the improvement of throughput and reduction in cost of DNA synthesis and sequencing technology [15,16,19]. Compared to *in vitro* DNA storage, *in vivo* storage takes advantage of the efficient cellular machineries of DNA replication, proofreading and long-chain DNA maintenance, offers the chance for assembly-free random access of data [18], and supports live recording of biochemical events *in situ* in living organisms as a generalized concept of information storage.

The development of synthetic biology and gene editing technologies have allowed us to change genetic information with unforeseen flexibility and accuracy [68,69]. Natural and engineered DNA targeting and modifying enzymes can be used as write modules in DNA storage systems, and the toolbox of DNA writers is rapidly expanding and improving in terms of programmability and accuracy [68–73]. The work of Shipman *et al.* offers an example for large-scale *in vivo* DNA storage. A library of indexed short DNA fragments encoding 2.6 KB of information was distributively inserted into the CRISPR arrays of multiple live bacterial genomes in a heterogenous population. For complete information retrieval, DNA from different cells was collected and sequenced, and the original information is reconstructed by proper alignment [74]. Yang *et al.* stored a total of 1.375 Bytes of information in the *E. coli* genome by different integrase enzymes [75]. Bonnet *et al.* used recombinases to write and erase information in living cells [76].

DNA writers can be broadly categorized into precise and pseudorandom writers on the basis of the mutational outcomes [68]. Precise DNA writers, including site-specific recombinases [72], reverse transcriptases [77] and base editors [78], generate predetermined mutations, whereas pseudorandom DNA writers, including site-specific nucleases [79–81] and the Cas1–Cas2 complex [79], generate targeted but stochastic mutations.

Site-specific recombinases are a class of highly efficient and accurate DNA writers that can flip, insert or excise a piece of DNA between their cognate recognition sites. Using recombinases, the information is heritably stored in a specific genomic location [72,75,80]. On top of this, reversible writing of information can be achieved by adding another enzyme (the excisionase), which erases the previously written information and resets the state of DNA [76]. The second class of precise DNA writers relies on reverse transcriptases [68,77]. For example, the SCRIBE (Synthetic Cellular Recorders Integrating Biological Events) system is activated in response to a specific stimulus (such as a chemical), producing a programable DNA sequence change [82]. The third class performs nucleotide-resolution manipulation of DNA via base editing [68,78], such as CAMERA (CRISPR-mediated analog multi-event recording apparatus) [83], generating deoxycytidine (dC)-to-deoxythymidine (dT) or deoxyadenine (dA)-to-deoxyguanine (dG) mutations.

Pseudorandom DNA writers relies on targeted double-stranded DNA breaks generated by site-specific nucleases [68], including Cas9, ZFNs and TALENs [79–81]. However, the write efficiency is highly dependent on the nonhomologous end joining pathway, which is lacking in many model organisms [79–81]. A second class of pseudorandom DNA writers leverages the cellular immune functionality of the Cas1–Cas2 system, which integrates information-encoding short ssDNA fragments (approximately 20–30 bp) into the CRISPR array in an oriented fashion [84].

For *in vivo* DNA storage, it is essential to consider the maximal amount of information that a single cell can carry. At present, *E. coli* is the most thoroughly studied prokaryote, but other microorganisms might be used for DNA storage as well. In an interesting example, Mitsuhiro *et al*. cloned the 3.5-Mb genome of the photosynthetic bacterium *Synechocystis* PCC6803 (3.5 Mb) into the 4.2-Mb genome of *Bacillussubtilis* 168, producing a 7.7-Mb chimeric genome [85]. This suggests a surprisingly large tolerance of prokaryotic cells in foreign DNA. If a cell can hold 4 Mb of DNA, it is possible to store 8 Mbit, or 1 MB, of information. In this scenario, a homologous recombination system handling long DNA fragments works more efficiently than a CRISPR-based system dealing with short fragments.

However, incompatibility and interactions between the information-carrying DNA and the host DNA pose challenges for *in vivo* DNA storage. For example, when Mitsuhiro *et al*. attempted to insert the exogenous genome into the genome of *B. subtilis*, efficiency was significantly affected by the host genome's symmetry [85]. As far as biosafety is concerned, although artificially encoded DNA is not prone to forming open reading frames, misexpression may emerge as the storage volume rises, and its biological consequences should be subject to close scrutiny. On the other hand, there is not enough evidence to show whether the insertion of DNA fragments affects the host cell's own gene expression. In eukaryotic cells, the problem is further complicated by the presence of a wide range of *cis*-acting elements. Effective methods must be devised to prevent the potential biological impacts associated with the insertion of DNA fragments carrying non-biological information.

## THE FUTURE OF DNA STORAGE

### Prospects and challenges

Although DNA information storage has enormous application potential, many problems need to be addressed before its broader implementation. First, the cost of writing and reading information is still prohibitively high and the efficiency of storing data is too low. However, DNA synthesis and sequencing costs have been reduced by 10-million-fold over the past 30 years, and the trend will continue to meet the needs of practical DNA storage in the foreseeable future [49,51]. It is predicted by the Molecular Information Storage Program that DNA synthesis cost will reduce to $10^−10^/bp by 2023 [86]. At the same time, the read and write speeds have gradually increased. In their original study (2012), Church *et al.* concluded that DNA synthesis and sequencing technologies require improvements of 7–8 and 6 orders of magnitude, respectively, to compete with current information read and write speeds [15]. The data presented by Goldman *et al.* show that the main contributor to the cost of DNA storage is synthesis and, based on their calculations, if the cost of synthesis is reduced by another 2 orders of magnitude (compared to 2013), DNA storage will outperform magnetic medium storage for decade-long data storage—a goal that could be achieved in just a few years [16]. In 2017, Erlich *et al.* gave a cost of $3500 per MB—about a quarter of the cost estimated by Goldman *et al.* [19], but they expected to use a more cost-effective approach for DNA synthesis as they developed a powerful error-correcting algorithm that tolerates base errors and losses. Very recently, Lee *et al.* showed a proof-of-principle enzymatic DNA synthesis scheme, which did not achieve single-base precision, but was still sufficient for complete information retrieval and showed a strong cost advantage over traditional phosphoramidite synthesis [37]. In addition, this synthesis scheme also supports a larger storage volume (∼500 to several thousand bases per synthesis) at a higher speed. However, in their implementation, the amount of data stored was extremely limited (144 bits) and whether this approach can be scaled up remains to be tested. Advanced coding and decoding algorithms may ultimately lift the technical requirements on synthesis and sequencing and enable production-grade DNA storage. In addition, storage-specific read and write methods may be developed outside the current synthesis and sequencing frameworks. Writing by the massive assemblage of premade oligonucleotides in a way similar to movable-type printing, for example, has recently been claimed to reach a 1 TB/day storage speed.

Random access is another function necessary for information storage purposes. PCR is typically performed using specific primers to obtain selective information stored in DNA. For long-chain DNA storage, PCR with appropriate primers upstream and downstream of the desired information will suffice. However, for oligo DNA storage systems, the entire library needs to be sequenced and assembled before fragmental information can be acquired. Based on powerful error correction codes and algorithmic design, Organick *et al.* developed a framework to minimize the amount of sequencing required to obtain specific data in an oligo library [18]. They managed to retrieve 35 files (with a total size >200 MB) independently without errors. According to their estimates, the method could be extended to an oligo library with a few TBs of storage capacity. It is worth mentioning that the work of Organick *et al*. is also an attempt to store the largest amount of data in DNA molecules so far (at the time of writing in 2019).

Finally, techniques to erase and rewrite information in DNA remain to be developed. Existing DNA storage methods support one-time storage only and thus are suitable for information that does not need to be modified, such as government documents and historical archives. However, the continuous development of synthetic biology has shown the possibility of solving this problem. Artificial gene circuits with stable DNA encoding functions have been designed [70–73,78–81]. For example, using a ‘Set’ system of recombinant enzymes and a ‘Reset’ system of integrase and its excision partner, a controllable and rewritable switch could be implemented [76].

### Carbon-based storage

Thanks to the rapid development of DNA manipulation technologies, DNA has become a promising new storage medium. However, other types of polymers may also be used in the field of information storage. Most of them are organic polymers, which, together with DNA molecules, constitute a novel carbon-based storage system different from traditional silicon-based storage.

Like DNA, proteins are an indispensable class of molecules in living systems. Their heterogeneous composition shows potential usage for information storage. However, such attempts are currently focused on the state of the protein rather than its amino acid sequence. For instance, a protein adopting two different states may encode 0 and 1, and information may be stored by switching and stabilizing the states by specific means. A typical example is a photo-switchable fluorescent protein, which changes color when absorbing photons of a particular wavelength [87,88]. Despite its high controllability, the information density is limited to 1 bit per molecule.

In theory, any heterogeneous polymer may serve the purpose of information storage as long as its component monomers can be handled with precision. Current attempts include DNA template guided incorporation of nucleic acid derivatives or small peptides into self-replicating biopolymers [89–91]. In recent years, the discovery of six non-natural nucleic acids that are able to form stable DNA duplex structures and even carry on genetic information suggests their use for DNA storage [92,93]. In addition to biopolymers, the synthesis of high-molecular-weight polymers such as polyamides and polyurethanes by precise sequence control methods has also been reported in many studies [94–96]. Unfortunately, the read and write techniques for these polymers are far less mature than DNA synthesis and sequencing at the present time. For example, sequencing of synthetic polymers relies on more general analytical methods such as MS/MS and NMR [97–99]. Interestingly, single-molecule nanopore sequencing is expected to be a powerful tool for reading information in synthetic polymers [100,101].

With more types of monomers able to be integrated, synthetic polymers may exhibit higher self-information and thus storage capacity. In addition, it may be more amenable to certain storage functions such as data erasure and rewriting. On a different scale, composite encoding has been applied to information storage. By using mixtures of nucleic acids or metabolites, one can potentially augment coding capacity in the continuous compositional space of components [102,103].

Taken together, synthetic polymers hold great promise for molecular information storage in non-living systems. With the development of sequence control and acquisition technologies, biological and synthetic polymers may form a new framework of carbon-based storage in the future and gradually replace traditional silicon-based storage systems in specialized or general applications.
